# Directed graph mapping shows rotors maintain non-terminating and focal sources maintain self-terminating Torsade de Pointes in canine model

**DOI:** 10.3389/fphys.2023.1201260

**Published:** 2023-07-26

**Authors:** Robin Van Den Abeele, Sander Hendrickx, Enid Van Nieuwenhuyse, Albert Dunnink, Alexander V. Panfilov, Marc A. Vos, Eike M. Wülfers, Nele Vandersickel

**Affiliations:** ^1^ Biophysics Group, Department of Physics and Astronomy, Faculty of Sciences, Ghent University, Ghent, Belgium; ^2^ Department of Medical Physiology, University Medical Center Utrecht, Utrecht, Netherlands; ^3^ Laboratory of Computational Biology and Medicine, Ural Federal University, Yekaterinburg, Russia; ^4^ World-Class Research Center “Digital Biodesign and Personalized Healthcare”, Sechenov University, Moscow, Russia

**Keywords:** directed graph mapping, cardiac arrhythmias, electrophysiological mapping, Torsade de Pointes, focal sources, reentry, chronic atrioventricular block

## Abstract

Torsade de Pointes is a polymorphic ventricular tachycardia which is as yet incompletely understood. While the onset of a TdP episode is generally accepted to be caused by triggered activity, the mechanisms for the perpetuation is still under debate. In this study, we analysed data from 54 TdP episodes divided over 5 dogs (4 female, 1 male) with chronic atrioventricular block. Previous research on this dataset showed both reentry and triggered activity to perpetuate the arrhythmia. 13 of those TdP episodes showed reentry as part of the driving mechanism of perpetuating the episode. The remaining 41 episodes were purely ectopic. Reentry was the main mechanism in long-lasting episodes (>14 beats), while focal sources were responsible for maintaining shorter episodes. Building on these results, we re-analysed the data using directed graph mapping This program uses principles from network theory and a combination of positional data and local activation times to identify reentry loops and focal sources within the data. The results of this study are twofold. First, concerning reentry loops, we found that on average non-terminating (NT) episodes (≥10 s) show significantly more simultaneous reentry loops than self-terminating (ST) TdP (<10 s). Non-terminating episodes have on average 2.72 ± 1.48 simultaneous loops, compared to an average of 1.33 ± 0.66 for self-terminating episodes. In addition, each NT episode showed a presence of (bi-)ventricular loops between 10.10% and 69.62% of their total reentry duration. Compared to the ST episodes, only 1 in 4 episodes (25%) showed (bi-)ventricular reentry, lasting only 7.12% of its total reentry duration. This suggests that while focal beats trigger TdP, macro-reentry and multiple simultaneous localized reentries are the major drivers of long-lasting episodes. Second, using heatmaps, we found focal sources to occur in preferred locations, instead of being distributed randomly. This may have implications on treatment if such focal origins can be disabled reliably.

## 1 Introduction

Torsade de Pointes (TdP) is a polymorphic ventricular tachycardia, first described by Francois Dessertenne in 1966 (Dessertenne, 1966). The particular name was given as Dessertenne observed a typical ‘twisting of the peaks’ of QRS around an imaginary baseline. Nowadays, TdP is more generally seen as any polymorphic ventricular tachycardia occurring in the setting of a long QT syndrome (LQTS) (Viskin, 1999; El-Sherif and Turitto, 2003). TdP is a dangerous arrhythmia, as it can degenerate into ventricular fibrillation and cause sudden cardiac death also in children and young adults (Vincent, 1998).

LQTS may be classified into either congenital or acquired. The prevalence of congenital LQTS is estimated to be close to 1 in 2000 individuals (Schwartz et al., 2009). There are 17 known subtypes of congenital LQTS, each associated with a different genotype (Wilders and Verkerk, 2018; Ponce-Balbuena and Deschênes, 2021). Drugs prolonging the QT interval are an active area of research, as this is one of the main reasons preventing new anti-arrhythmic drugs from entering the market (Laverty et al., 2011). Indeed, drug-induced TdP was the reason behind around one-third of all drug withdrawals between 1990 and 2006, even though an event of TdP was extremely rare (order of 1/100.000) (Shah, 2006). A recent overview of drug cardiotoxicity (including TdP) was published by Mamoshina et al. (2021).

Although TdP is thus an important arrhythmia, many aspects of its mechanism remain unclear. A long QT interval is caused by the prolongation of ventricular action potentials (AP) through a decrease in so-called ‘repolarization reserve’ at single cell level (Roden, 2006). Repolarization reserve, the redundancy of repolarizing forces, decreases either via a reduction of the outward potassium currents (‘loss-of-function’) or an increase in late entry of sodium or calcium currents (‘gain-of-function’) during the repolarization phase of the AP. A decrease in repolarization reserve (e.g., through an increase of L-type calcium channel expression) can ultimately even cause early afterdepolarizations (EAD) (Vandersickel et al., 2014).

Many studies reported that TdP episodes start with a focal beat, originating from groups of cells exhibiting EAD at the same time (Asano et al., 1997; Choi et al., 2002; el Sherif et al., 1996; El-Sherif et al., 1997; Kozhevnikov et al., 2002; Murakawa et al., 1997; Schreiner et al., 2004; Senges et al., 2000). Kim et al. (2015a); Choi et al. (2002) additionally reported that local elevations of cytosolic calcium concentration and overload of the sarcoplasmic reticulum can directly cause EAD and act as trigger of TdP in explanted rabbit hearts. In addition, it has been shown that a large heterogeneity of repolarization time can play a crucial role in the onset of a focal beat (Dunnink et al., 2017; Rivaud et al., 2020). Complementary to this research, Liu et al. (2019) performed *in silico* experiments and found that premature ventricular complexes always originated spontaneously from the steep repolarization gradient region. They named the mechanism R-from-T and suggest that R-from-T is a common mechanism underlying polymorphic ventricular tachyarrhythmia initiation in LQTS. The heterogeneity in repolarization time can be attributed to reported spatial heterogeneity in the expression of the ion channels involved in repolarization. The spatial heterogeneity of channel expression also exhibits sex differences, explaining higher risk of TdP in female animal models and women (Němec et al., 2019).

Contrary to the established mechanism of the trigger of TdP, the perpetuation of TdP remains controversial. In animal experiments, researchers claimed that focal mechanisms (Murakawa et al., 1997; Senges et al., 2000; Choi et al., 2002; Schreiner et al., 2004; Boulaksil et al., 2011; Kim et al., 2015b; Dunnink et al., 2017), non-stationary reentries (El-Sherif et al., 1997; Akar et al., 2002; Kozhevnikov et al., 2002; Rivaud et al., 2020), or both mechanisms (Asano et al., 1997; el Sherif et al., 1996; Boulaksil et al., 2011) are the basis of the perpetuation of TdP. In theoretical studies, it was claimed that the typical twisting ECG pattern may be due to the drift of a reentrant circuit (Abildskov and Lux, 1991; 2000; Gray et al., 1995; Winfree, 1995; Schmitt et al., 2001). For a focal mechanism on the other hand, the twisting of the ECG may be caused by multiple shifting foci generated by EAD (Sato et al., 2009; de Lange et al., 2012), or a competition between focal beats generated by fixed heterogeneities with reduced repolarization reserve (Vandersickel et al., 2016).

In previous research performed by some authors of this manuscript, needle experiments were conducted on the anaesthetized chronic atrioventricular block (CAVB) dog model, of which about 70% develop TdP upon a dofetilide challenge. We investigated 54 TdP episodes in 5 susceptible dogs, of which 45 were self-terminating (ST) and 9 were non-terminating (NT). It was found that short lasting episodes were purely maintained by focal activity, while 4 longer lasting (>14 beats) and all 9 NT episodes contained at least one reentry circuit (Vandersickel et al., 2017). Detection of those reentries was accomplished using a novel method called directed graph mapping [DGM; Vandersickel et al. (2019)]. DGM creates directed networks from excitation patterns of every time frame recorded. If a network contained at least one closed cycle, we marked that time frame as containing a reentry. There are two types of reentry, functional reentry, also called rotors, or anatomical reentry, where a wave rotates around an inexcitable obstacle.

Although DGM provided new insights into mechanisms of TdP, our previous study was not able to determine the type of reentry (functional or anatomical), the number, the location, and whether rotors meander (Nayyar et al., 2017). It was also not studied whether focal sources were homogeneously or heterogeneously distributed and how many focal beats occurred during a TdP episode.

Therefore, we extended DGM with the ability to better analyse each TdP episode. DGM is now able to identify and distinguish between multiple simultaneous reentries during a TdP episode. As a result, we were able to distinguish between ‘holo-ventricular’ macro-reentries (circling one or both cavities) from other, localized reentries, while also tracking the number of reentries present. We were thus able to compare these features between ST and NT groups In addition, triggered activity was further investigated by tracking the locations of all focal sources and putting them into spatial heatmaps for discovering the nature of their distribution. This information was then used to determine whether the focal sources have preferred locations.

## 2 Methods

### 2.1 Animal model and data collection

The experimental procedures were conducted in accordance with the guidelines drawn up by the European Community for the use of experimental animals (EU Directive 2010/63/EU). Each operation was completed with the approval from the Committee for Experiments on Animals of Utrecht University.

This research used data collected from a total of 54 TdP episodes divided over 5 dogs. 4 dogs were female (dogs 1, 2, 3, and 5), while 1 dog was male (dog 4). Each dog was premedicated 30 min before the procedure (0.02 mg/kg atropine i. m., 0.5 mg/kg methadone i. m., 0.5 mg/kg acepromazine i. m. and 0.1 mg/kg meloxicam i.m.), then anaesthetized by injecting sodium pentobarbital (25 mg/kg i.v.). Subsequently, anaesthesia was maintained by isoflurane 1.5% in a 2:1 mixture of N_2_O:O_2_ via mechanical ventilation at 12 breaths/minute. Ampicillin (1,000 mg) was administered before (i.v.) and after (i.m.) the AV-block procedure, while buprenorphine (0.3 mg, i.m.) was provided post-procedure. AV-block was induced by ablating the bundle of His through radiofrequency ablation (Timmermans et al., 2002).

Once the AV-block was in place, the dogs were allowed ≥6 weeks of ventricular remodeling before a dose of 25 μg/kg/5 min dofetilide was administered to induce episodes of TdP. If the duration of the TdP-episode exceeded 10 s, the heart was defibrillated and the episode was labelled as NT, as it reached the maximum allowed arrhythmia duration.

Cardiac excitation was measured by inserting 60 needles into the ventricular wall, each containing 4 electrode terminals spaced 0.4 cm apart. All electrodes recorded unipolar electrograms (ActiveTwo system, Biosemi, Amsterdam, the Netherlands). Two types of data were extracted: 1) The local activation times (LAT) and 2) the spatial location of the needle electrodes. Both were used by DGM to find reentries and focal sources.

### 2.2 Local activation time annotation

The LAT annotation method was previously published in Vandersickel et al. (2017). For the benefit of the reader, we summarize the procedure:

Recorded unipolar electrograms were annotated where their derivative was most negative within 200 ms. Additionally, within ±10 ms of each such annotation, electrogram amplitude had to be greater than 10% of its average peak amplitude. Points that did not meet these requirements were discarded, unless one of their neighbouring electrodes had an LAT at a time close enough to the LAT of the point in question.

Subsequently, the points were visualized and manually removed and added at times when the automatic algorithm had failed. This modification was thoroughly checked by two researchers from different departments (Ghent and Utrecht) to reduce bias.

### 2.3 Positional data derivation

**FIGURE 1:**
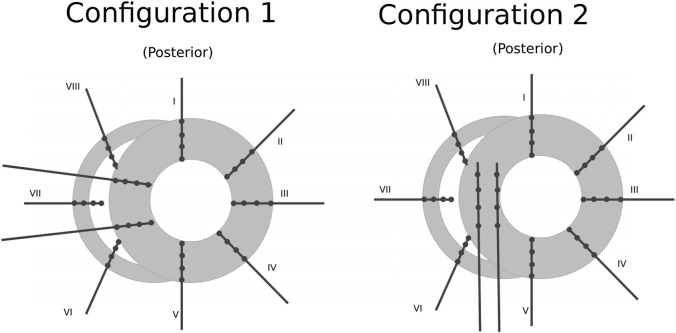
Schematic representation of the positioning of the needles (black lines) and their electrode terminals (black dots) inside one layer of the ventricular wall (gray area). Configuration 1 was used for dog 1 and configuration 2 for dogs 2–5. The whole ventricles are represented by stacking 6 of these layers on top of one another spaced 1.2 cm apart. Figure reproduced from Vandersickel et al. (2017).

The positional data of the electrodes is a necessary input for DGM. During the procedure, the heart was visually divided in 6 layers, each with a thickness of 12 mm. In each layer, 10 needles were inserted into the ventricular wall according configuration 1 (dog 1) or configuration 2 (dogs 2–5) as shown in Figure 1. Each needle contained 4 electrode terminals, spaced 4 mm apart. While some relative distances between electrodes were known, a visual estimation had to be made to obtain the absolute positions based on the configurations in Figure 1.

### 2.4 Directed graph mapping

DGM (Vandersickel et al., 2019; Van Nieuwenhuyse et al., 2021) is an algorithm based on network theory developed to detect reentry loops (both rotors and anatomical reentry) and focal sources in electroanatomical maps of the heart. DGM uses LAT and electrode coordinates to create a network of unidirectional arrows representing the propagation of the electrical signals in the heart. A full description of the algorithm can be found in Van Nieuwenhuyse et al. (2022) or at https://dgmapping.ugent.be. An abridged version of the basic principles is given in the next paragraph.

The algorithm generally works as follows: Consider a network with a group of *N* electrodes {*E*_*n*_ *∀n* ∈ [1, *N*]}, created at time *t*. For an arrow to be drawn between two neighbouring electrodes *E*_*x*_ and *E*_*y*_, the conduction velocity between the electrodes is calculated by dividing the distance between their locations *Δd* by the difference in LAT (*ΔLAT*). If this velocity is within an interval of permissible values (*CV*_min_ and *CV*_max_), an arrow is drawn from the electrode with earlier LAT to the other.

$$CV_{\text{min}}<\frac{\Delta d}{\Delta LAT}<CV_{\text{max}}$$ (1)

The determination of the neighbours is explained in detail in the Supplementary Material S1.1.

**FIGURE 2:**
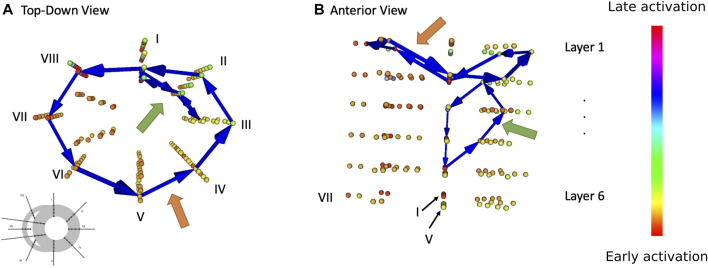
Result of DGM analysis during one episode of TdP in dog 1. Every coloured sphere represents the location of a needle electrode (cf. configuration 1 from Figure 1) Blue arrow show loops detected by DGM. Orange arrows point to a bi-ventricular loop, green arrow to a localized re-entry. **(A)** Top-down view. **(B)** Anterior view.

A graph resulting from the above procedure cannot yet contain loops, even if there exists one (patho-)physiologically: Between at least two points of a reentry or loop, there would be a high LAT next to a low LAT with their difference equalling the cycle length of the loop. Thus, after constructing a first graph, a second one is created at a time *t* + *Δt* in exactly the same manner and then merged with the first. Since the LAT discontinuity exists now at a different part of the loop, reentrant activity can be identified from closed cycles of arrows in the merged graph. All parameters used for this process are described in the Supplementary Material S1.2. Finally, we excluded the loops that represented an impossible trajectory, namely, loops containing two arrows crossing one-another, and loops for which the angle between two consecutive arrows was too acute (i.e., when the cosine of the angle was larger than 0.75.). An example for a DGM result can be found in Figure 2, where two loops were found during an episode of TdP. For an example of detected focal sources, see Supplementary Figure S3.

### 2.5 Grouping

After filtering, multiple cycles were found to represent the same reentry. These similar loops were grouped together and condensed into one, using an unsupervised machine learning technique called Density-based spatial clustering of applications with noise (DBSCAN; Ram et al. (2010); Birant and Kut (2007)). This resulted in a final number of distinct reentry loops at any point in time. DBSCAN was chosen because it is one of the few clustering algorithms that did not need an advance input parameter of how many clusters are present. On top of this, the algorithm required just 2 parameters to fine-tune: the *ϵ*-distance (*d*_*Eps*_) of the neighbourhood *N*_*Eps*_(*p*) for all points *p* and the minimum number of points in a cluster *minPts*. This neighbourhood is defined by Eq. 2 for the remaining points *q* in the dataset and represents the influence of point *p* to be in the same cluster as point *q*. We set this parameter (*d*_*Eps*_) to 10 mm and the parameter *minPts* to 2, by manually testing different values on a subset of the data.

$$N_{\mathrm{Eps}}\left(p\right)=\left\{q\in D|dist\left(p,q\right)\leq d_{\mathrm{Eps}}\right\}$$ (2)

**FIGURE 3:**
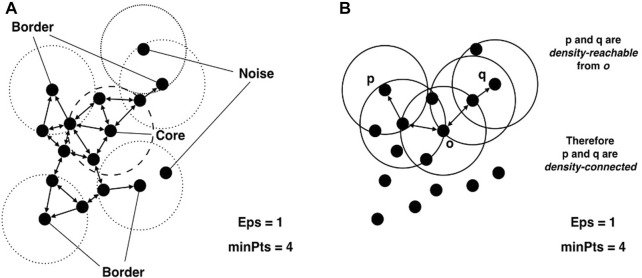
Visual explanation of **(A)** core, border and noise points and **(B)** density reachability. This figure is reproduced from Hahsler et al. (2019) under CC-BY licence.

A point *p* gets classified into (Hahsler et al., 2019) 3 classes. A visualization of this classification is shown in Figure 3A.

• *Core point*: if the neighbourhood of *p* contains enough points |*N*_*Eps*_(*p*)| > *minPts*.
• *Border point*: If p is not a core point, but is in the neighbourhood of a core point.
• *Noise point*: Otherwise.

A point *p* is then *directly density-reachable* to a point *q* if.

• |*N*_*Eps*_(*p*)| > *minPts* and
• *q* ∈ *N*_*Eps*_(*p*).

A point *p*_1_ is then *density-reachable* from point *p*_*n*_ if there exists a sequence of points (*p*_1_, *p*_2_, *…* , *p*_*n*_) where *p*_*i*_ is directly density-reachable from *p*_*i*+1_. A visualization of this concept is shown in Figure 3B.

A cluster *C* is then defined as a subset of points where.

• If *p* ∈ *C* and q is density-reachable from *p* then *q* ∈ *C*.
• *∀p*, *q* ∈ *C*, *p* is density-reachable to *q*.

### 2.6 Triggered activity

Focal activity was detected in the directed graph by identifying nodes with only outgoing arrows (example visualization in Supplementary Figure S3). When such nodes were found, the corresponding electrode was considered a candidate focal origin at the time of its LAT. Subsequently, for all candidates, it was manually verified whether the unipolar electrogram had no noticeable upstroke, indicating that there was no incoming wave in that location. A second check was carried out by inspecting the sequence of LAT in a 3D visualization program.

All sources were then aggregated for each dog and placed into spatial heatmaps. These heatmaps were checked for preferred focal locations or for random behaviour. This randomness should manifest itself as a truncated binomial distribution according to Eq. 3 with a total number of *n* focal sources and *e* = *p* ^−1^ electrodes. *p*_*x*_ is the chance for a position to contain *x* focal sources. When a distribution deviates significantly from the norm, we can conclude that there are preferred locations for the focal sources to occur.

$$p_{x}=\frac{n!}{x!\left(n-x\right)!}p^{x}{\left(1-p\right)}^{n-x}$$ (3)

## 3 Results

### 3.1 Recorded data and previous analysis

As previously published, 54 TdP episodes were successfully initiated in 5 susceptible dogs (Vandersickel et al., 2017). Example ECG signals of an ST and NT episode can be seen in Supplementary Figure S4. As in the previous publication, our improved version of DGM detected reentry in 13 of the 54 episodes, with 9/13 NT and 4/13 ST episodes. The remaining 41 episodes showed no sign of reentry as the driving mechanism for TdP.

We performed the following additional analyses: First, all episodes were analysed for reentry loops with the current version of DGM. We quantified the number of reentry loops during each episode and how these loops evolve over time. In addition, we investigated if ‘holo-ventricular’ reentry was present. We define holo-ventricular reentries as loops that traverse the ventricular walls such that describe closed loops around ventricular cavity. Additionally, a bi-ventricular reentry is defined as a loop encircling both cavities in one rotation (i.e., similar to a holo-ventricular loop but not closing through the ventricular septum). Unfortunately, the sparsity of the dataset did not allow us to distinguish between functional and anatomical reentry for the localized loops. Second, we also investigated the focal sources as described in the methods section. We created heatmaps to uncover whether the focal sources have preferred locations in all episodes. Subsequently, the heatmaps for the separate episodes were combined into one a single heatmap per dog and evaluated for randomness.

#### 3.1.1 Detected episodes of Torsade de Pointes

The two different types of reentrant TdP episodes (ST and NT) were compared to one another on two different areas. First, we looked at the number of distinct reentry loops that were present at the same time. Second, we analysed presence and duration of holo- and bi-ventricular reentry. Figure 2 shows exemplary loops detected by DGM in one needle configuration at one time step during an episode of TdP.

We did not find a correlation between QT intervals (or their variance) and NT or ST episode propensity in individual dogs (cf. Supplementary Figure S5).

#### 3.1.2 Number of reentries

For each reentry episode, the detected loops were grouped together into distinct reentries (Section 2.5). The number of simultaneous reentries were subsequently compared between the ST and NT group. Note that only episodes with at least one reentry present are considered here (9 NT and 4 ST spisodes).

**FIGURE 4:**
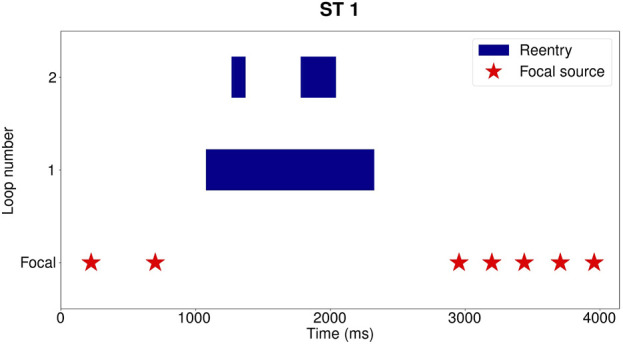
Example for reentry episode Dog 1: ST 1, taking place between 606 s and 611 s after administration of dofetilide. Each blue block represents one distinct reentry loop. In the row underneath the reentry, the start of the focal sources are indicated with red stars. Three distinct reentries were detected between 1,000 ms and 2,400 ms, with at most 2 concurrently present. One loop is present from 1,000 ms to 2,400 ms, a second reentry from 1,300 ms to 1,450 ms and the last one from 1800 ms to 2,100 ms. Following the reentry, 5 more focal beats are detected before termination of the episode.

A demonstration of this procedure is shown in Figure 4. Each red star represents the start of a focal beat, while a reentry takes the form of a blue block. It is apparent in Figure 4 that the episode starts with the emergence of 2 focal beats. The first beat was detected at 200 ms and the second at 700 ms after the start of the episode. Between 1,000 ms and 2,400 ms, reentry was detected by DGM. After the termination of the reentry, 5 more focal beats were detected before the episode terminates.

**FIGURE 5:**
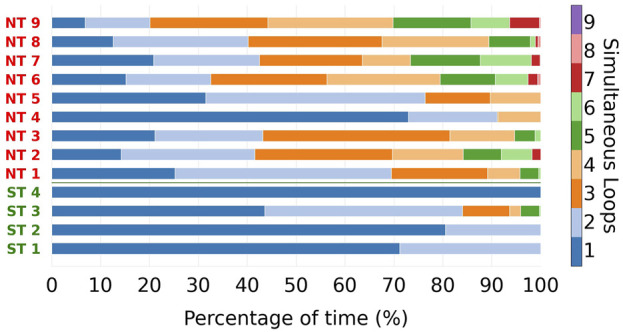
For every reentry episode, the total reentry duration was split into parts, based on the number of simultaneous reentry loops present. These parts are indicated with different colours. The designations of the ST episodes are coloured green, of NT episodes in red. NT episodes reach higher numbers of simultaneous loops compared to the ST group. Average and maximum number of simultaneous loops during the episodes were significantly different between NT and ST groups.

For each episode, we determined duration and time at which a certain number of distinct reentries were detected. These durations were converted into percentages, where 100% is the total time at which reentry is present during an episode. The outcome is presented in Figure 5, with each colour representing a different number of distinct simultaneous loops.

**FIGURE 6:**
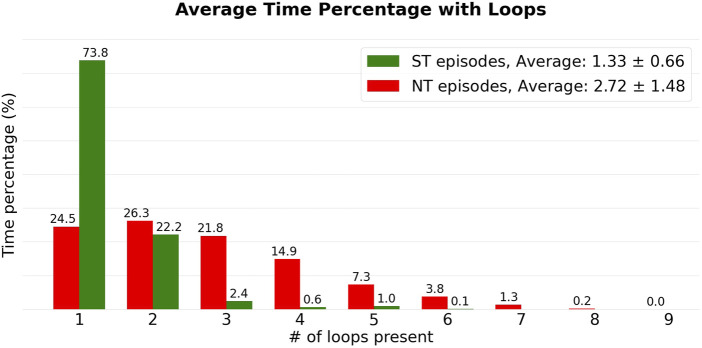
The episodes from Figure 5 are added to their respective groups (NT: green, ST: red). Subsequently, the averages were calculated for each colour in Figure 5, corresponding to a certain number of simultaneous loops. This resulted in the heights of the bars.

From Figure 5, we can observe that ST episodes have a lower number of simultaneous reentries than NT episodes. To better highlight this, we divided the different episodes in two groups: ST and NT. For each colour in Figure 5 the average was taken, resulting in an average composition for ST and NT reentry episodes regarding the number of reentries (see Figure 6).

From Figure 5, we observe that the number of simultaneous reentries is generally lower in ST episodes. The most notable discrepancy is the time during which only a single loop was detected. This was observed on average during 73.8% of the time in the ST group, compared to the NT group which was only 24.5%.

**FIGURE 7:**
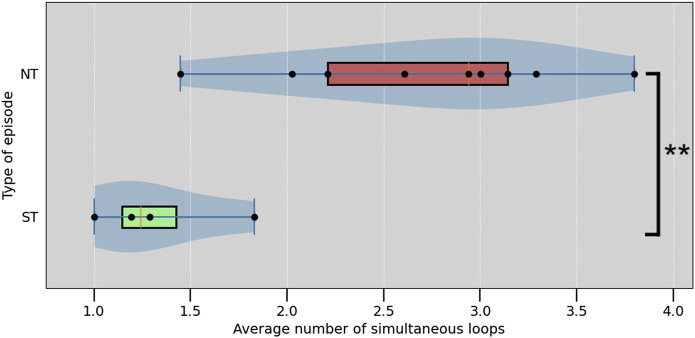
Box plot of the average number of simultaneous loops in each episode. ** indicate a significant difference with *p* <0.01 (Mann–Whitney *U* test, $p\dot{=}0.003$ ).

Looking at the averages, ST episodes have an average of 1.33 ± 0.66 (average ± standard deviation) simultaneous loops, while the NT group has an average of 2.72 ± 1.48 loops. The average number of simultaneous loops for are shown in Table 1. Both, the average number of loops present, and the maximum number of simultaneous loops during an episode were significantly different between ST and NT, determined using a Mann—Whitney *U* test ( $U=1.0,p\dot{=}0.003$ and $p\dot{=}0.007$ , respectively). The average numbers of simultaneous loops compared between ST and NT are also shown in Figure 7.

**TABLE 1:** Average number of simultaneous reentry loops per TdP episode, ordered by non-terminating (NT) or self-terminating (ST).

NT	2.21	3.00	2.61	1.45	2.02	3.29	3.14	2.94	3.80
**ST**	1.29	1.19	1.83	1.00					

#### 3.1.3 Holo-ventricular reentries

**FIGURE 8:**
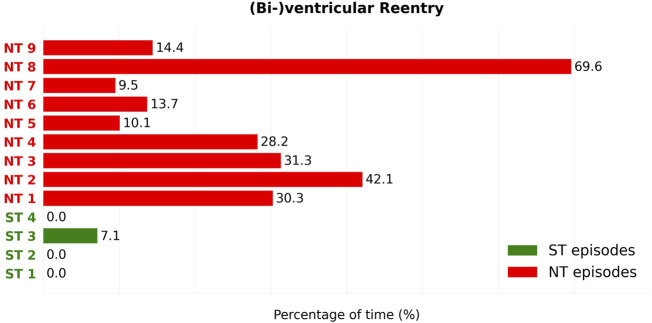
For each reentry episode, the percentage of time that a reentry proved to be either holo- or bi-ventricular is observed. The duration percentages of the ST (Green) and NT (Red) episodes can subsequently be compared. We can see that this type of reentry is barely present in the ST group (Green), only in 1 of the 4 episodes. In contrast to the ST group, all 9 NT episodes show presence of this type of reentry. In addition, all NT episodes developed (bi-)ventricular reentry to a higher degree that any episode in the ST group. The percentages of time during which holo- or biventricular reentry is present are significantly different between ST and NT groups (Mann–Whitney *U* test, $p\dot{=}0.024$ ).

For each episode, the duration of time was measured during which a reentry loop enclosed either one (holo-) both of the ventricles (bi-ventricular reentry). Again, only episodes featuring at least one reentry was present (but not necessarily a holo- or bi-ventricular reentry). Figure 2 exemplarily shows the path of a bi-ventricular reentry (orange arrow). This duration is displayed in percentages in Figure 8.

Of the ST episodes, only 1 in 4 cases showed holo-ventricular reentries. Holo-ventricular reentries were detected on average 1.78% of the time during reentry in ST episodes. In contrast, all 9 NT episodes showed the presence of holo-ventricular reentry to a greater extent than any ST episode. This duration ranged from 10.10% to 69.62% of their total reentry duration, resulting in an average 27.7% of the reentry duration containing holo-ventricular reentries.

### 3.2 Focal beats

For each dog, heatmaps were created containing all the focal locations detected during all recorded TdP episodes in that dog (Supplementary Figures S6–S10). They were subsequently converted into bar charts where the electrodes are collected into bars based on the amount of focal sources they contain. These values are then compared to a completely random distribution of sources over the same number of electrodes, resulting in a truncated binomial distribution (Eq. 3).

**FIGURE 9:**
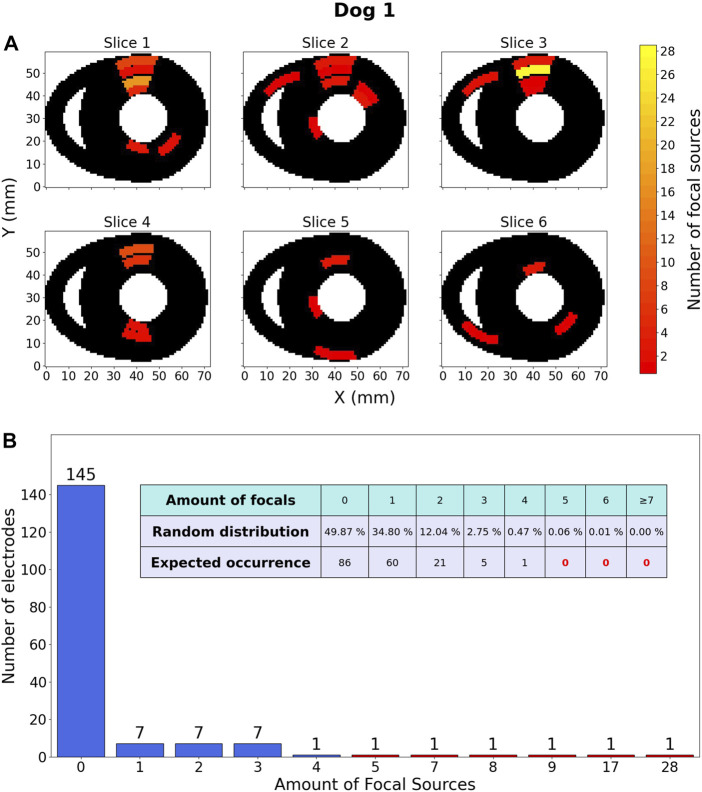
Focal source location results within one dog. **(A)** Heatmap, representing the ventricles of dog 1 in which 120 focal sources were discovered over 18 TdP episodes, including those that degenerated into reentry. These sources are distributed over a total of 173 electrodes. The colour of an area indicates the amount of focal sources. The Y-axis is oriented from the anterior wall (*Y* =0) to the posterior wall (*Y* =60). **(B)** Barchart counting all electrodes (y-axis) containing a certain amount of focal sources (x-axis). The embedded table shows the expected distribution of amount of focal sources if they would be randomly distributed.

The distribution for dog 1 is shown as an example in Figure 9. The other distributions for dogs two to five were qualitatively similar. Figures for the other 4 dogs can be found in the Supplementary Materials (Supplementary Figures S7–S10).

#### 3.2.1 Description of focal distribution

The most important observation of Figure 9 is the fact that according to the random distribution it should be almost certainly impossible for an electrode to be at the origin of 7 or more focal beats. However, the data showed 5 electrodes in this category (red bars), indicating that focal sources are not uniformly distributed. Another notable observation is that for a random distribution, it is expected to have 49.87% of all electrodes without any focal activity. However, the data shows 145/173 (83.81%) electrodes detecting no focal sources.

The 4 other dogs show qualitatively similar results. All comparisons show a consistent disparity when comparing the data to the random model, meaning that all heatmaps show some hotspots of focal activity of which the accompanying random distribution tells us they are almost certainly impossible.

## 4 Discussion

### 4.1 Reentries and focal sources

Using DGM, we were able to observe multiple simultaneous reentries maintaining TdP in the CAVB dog model. Moreover, we found that there were differences in the types of loops between the ST and NT groups.

First, comparing the number of simultaneous reentries, Figure 6 reveals a difference between the ST and NT groups. On average, ST episodes were found to have less simultaneous reentry loops, compared to the NT group. Only 1 of the 4 ST reentry episodes maintained more than 2 loops at the same time. In the NT group, all 9 TdP episodes showed 4 or more simultaneous loops at some point during the episode. Therefore, we suggest that NT reentry episodes may involve more concurrent loops and therefore exhibit more chaotic behaviour. We speculate that this chaos could be caused by the breaking-up of the initial rotating waves (anatomical or functional), making it more difficult for all reentrant activity to self-terminate. This could explain why NT episodes indeed do not stop spontaneously. This theory is strengthened by Dharmaprani et al. (2022), in which the authors discovered self-terminating episodes of VF distinguishing themselves from non-terminating episodes by a trend toward slower phase singularity (PS) destruction, slower rates of PS formation, and a slower mixing rate of the VF process. Smoczyńska et al. (2021) showed that there could be a connection between the spatial dispersion of the repolarization and the emergence of reentry. It would be interesting to see if the degree of this dispersion correlates to the number of reentries present.

Second, we found a difference in the presence of holo- or bi-ventricular reentry (Figure 8). All 9 NT episodes develop a significant presence of either holo- or bi-ventricular reentry, opposed to only 1 of the 4 ST episodes. In addition, this (bi-)ventricular reentry in the ST episode was present to a significantly lesser degree than in all 9 NT TdPs, indicating that this type of reentry can be another possible driving mechanism for the perpetuation of TdP in CAVB dogs.

It was argued by Nayyar et al. (2017) that observed reentries might actually be ‘pseudo-reentries’ due to possible lines of block. In this study, we can confidently rule out pseudo-reentries because our rotor detection algorithm does not rely on phase singularities that can be present at lines of block. Moreover, as we have shown in our previous study, the average time between subsequent activations of the same electrode is significantly smaller during reentries than in case a focal beats (Vandersickel et al., 2017). Nayyar et al. (2017) also observed that several factors (mass of viable tissue, number and synchronicity of neighbouring wave fronts, local conduction velocity, anisotropy, and recording distance) determine the amplitude of such pseudo-reentries in a unipolar electrogram. Ensuring that no upstroke was present in the local electrogram during detected focal beats, we were able to distinguish with some certainty focal beats from reentries. Therefore, our evidence points to reentry in the longer lasting cases, whereby the number of simultaneous reentry loops is increased for the NT episodes, possibly due to wavebreaks as suggested by Dharmaprani et al. (2022).

### 4.2 Spatial distributions of triggers/focal sources and therapeutical utility

This study also investigated the locations of the focal sources in the CAVB dog model. Heatmaps for all 5 dogs (Supplementary Figures S6–S10) clearly show that the detected focal beats originate from preferred locations within the ventricles. One explanation for this phenomenon can be that there are heterogeneities within the tissue containing a reduction in repolarization reserve compared to the surrounding tissue. Upon a global reduction in repolarization production, these heterogeneities are able to produce focal beats. This mechanism has been demonstrated in a previous simulation study (Vandersickel et al., 2016). In addition, Dunnink et al. (2017) also suggested that heterogeneities could play a role in determining the locations for focal sources. A previous study from Dunnink et al. (2017) showed TdP in CAVB dogs initiating in regions of maximal spatial dispersion of repolarization. As TdP episodes always originate with a focal beat, this hypothesis seems to agree with our result of preferred locations of the focal sources.

Weiss et al. (2010) found that synchronization of chaotic EAD creates macroscopic ‘EAD islands’. When EAD are potent enough to trigger an AP from an EAD island, focal activity may emerge from those islands. The study further suggests that a twist of peaks as characteristic for TdP can be induced by pacing the ventricle at two separate sites of those EAD islands. In addition, Kim et al. (2015b) investigated a transgenic rabbit model of LQT1 and found that foci that initiated the TdP-episode were found mostly in the right ventricles, supporting our claim of preferred locations. During TdP however, multi-focal sites were found to be rather complex.

If, however, investigations in humans also indicate preferred focal origins, these locations could prove important ablation targets for patients suffering from LQTS. Indeed, Steinfurt et al. (2021) successfully performed catheter ablation of focal source locations in a short-coupled variant of TdP. Similarly, Pappone et al. (2023) found localized structural electrophysiological abnormalities in high-risk LQTS patients and ablation of these abnormal locations prevented recurrence of ventricular arrhythmias.

### 4.3 Limitations

An obvious limitation of this study was the low number of dogs studied (5). We did not study inter-individual differences but instead pooled the recorded TdP episodes across all dogs. Focal source localization was studied per dog, due to the likelihood of underlying anatomical or pathological causes.

The exact locations of needle electrodes were not recorded and had to be estimated visually. This could have an impact on the networks generated by DGM due to the calculation of conduction velocities (cf. Eq. 1) and therefore in-/exclusion of conduction paths. In future experiments, we recommend measuring the exact positions of the needles. This can be accomplished, e.g., with the use of a CT-scan. As this type of experiments requires to sacrifice the dogs, knowing the exact positions could allow to maximize the information which can be extracted from these terminal experiments.

The spacing between needle electrodes is fairly large. Especially along the epicardium of the ventricles and in the septum, the electrodes are spaced far apart. A denser and more uniform distribution of electrodes could increase accuracy in determining locations of the focal sources. It would also improve distinction between functional and anatomical reentry, which was not possible in the study (Nayyar et al., 2017). However, the spatial resolution was sufficient to distinguish between rotational activity and focal activity. In our previous study, we showed that the silent time (time with no activation) and the amplitude of the signals added additional evidence to differentiate between focal beat and reentry circuits (Vandersickel et al., 2017).

The algorithm applied for the clustering of loops is only sufficient for use in a general comparison between different episodes from the same kind of data as we did in this research. However, it is too naive to used as an absolute measurement for the number of loops. In addition, only the locations of the central points of reentries were used in the algorithm.

In previous research on TdP, meandering spirals were suggested as a candidate for causing the characteristic ‘twisting of the peaks’ (El-Sherif et al., 1997; Akar et al., 2002; Kozhevnikov et al., 2002; Kawatou et al., 2017; Rivaud et al., 2020). Sadly, tracking meandering proved difficult in our experiments. In case of minor meandering, the electrode grid proved to be too sparse to detect those deviations. In more complex episodes, the combination of many simultaneous loops and the tendency for the locations of the spiral tips to make rather large jumps from one rotation to the next, made it impossible to correctly track the meandering of a spiral wave over time. For the same reasons, it was also impossible to distinguish anatomical (micro or macro) reentry from functional reentry.

## 5 Conclusion

We showed previously that rotors are likely the main drivers of NT TdP episodes. However, rotors also occur in some ST episodes. It has become clear in this research that there are two main differences between NT and reentrant ST episodes of TdP in the CAVB dog model: In terms of complexity, NT episodes show a larger number of simultaneous reentries compared to ST ones. In addition, looking at the type of reentry, NT TdP episodes show a significant presence of holo- or bi-ventricular reentry, while they almost never occur during ST episodes. These observations should be studied further to make sure that it is the case in human hearths as well. These findings suggest that macro-reentries and multiple localized reentries are the main mechanisms maintaining long-lasting episodes of TdP.

Focal sources in the CAVB dog model were found to have preferred locations. Should such preferred locations also exist in humans, they might be an important ablation target to prevent recurrence of TdP.

## Data Availability

The raw data supporting the conclusion of this article will be made available by the authors, without undue reservation.
