# Supplementary material for: Directed graph mapping shows rotors maintain non-terminating and focal sources maintain self-terminating Torsade de Pointes in canine model
Source: Front Physiol. 2023 Jul 26;14:1201260. doi: 10.3389/fphys.2023.1201260 (PMC10411729; doi:10.3389/fphys.2023.1201260)
Supplement: Supplementary file 1 [file DataSheet1.PDF]

Supplementary Material

1 SUPPLEMENTARY MATERIALS

1.1 Neighbouring Algorithm

For Directed Graph Mapping (DGM) to be able to detect loops, the neighbours for each electrode need to be determined. Since there were only 2 different needle configurations for all episodes of Torsade des Pointes (TdP), a fully customized neighbouring algorithm was created for this dataset. The set of rules the neighbours must satisfy were defined as follows:

- Electrodes, located on the same needle are neighbours if they lay one position closer to and one position further from the centre of the heart.
- Electrodes on adjacent needles are neighbours if their positions on the needle differ by a maximum of one.
- In configuration 2 the electrodes inside the septum at the base of the needle connect to the electrodes outside the septum as demonstrated in figure S2. The same structure applies for the electrodes at the tip of those needles, but mirrored around the horizontal axis.
- The two electrodes should be spaced no more than 1 vertical layer apart.

Figures S1 and S2 show two visual examples of how the electrodes were connected to each other using this customized neighbouring algorithm.

1.2 DGM: Parameters

DGM requires a number of input parameters to be set to refine the detected loops Van Nieuwenhuyse et al. (2021).

1. The minimum number of arrows to form a loop was set to 5.
2. The minimum and maximum conduction velocity (CV_{min} and CV_{max}) determine a lower and upper limit for the conduction velocity for an arrow to be drawn between neighbours. CV_{min} was set to 0.25 mm/ms. CV_{max} was set to 2.0 mm/ms.
3. Phase variance is a mathematical construct, representing the regularity of the detected cycles. Cycles with lower variances represent more concentric routes in the network. The maximum variance threshold was set to 0.4 in the analysis.
4. Inter-variability is a measure for the consistency of the conduction velocity in a loop. Loops with the same conduction velocity between excitations have low inter-variability. It was set to a maximum of 0.4 during the analyses of the data.
5. The merge jump δt, is the time-gap between the construction of the 2 merged networks as previously explained. It was set to 25 ms.

1.3 Triggered Activity

For each dog, heatmaps were created containing all their focal locations (Figures S6–S10). They were subsequently converted into bar charts showing the amount of electrodes (y-axis) that contain a certain

amount of focal sources (x-axis). The data is then compared to a random distribution, using the same amount of electrodes and focal sources.

2 SUPPLEMENTARY FIGURES

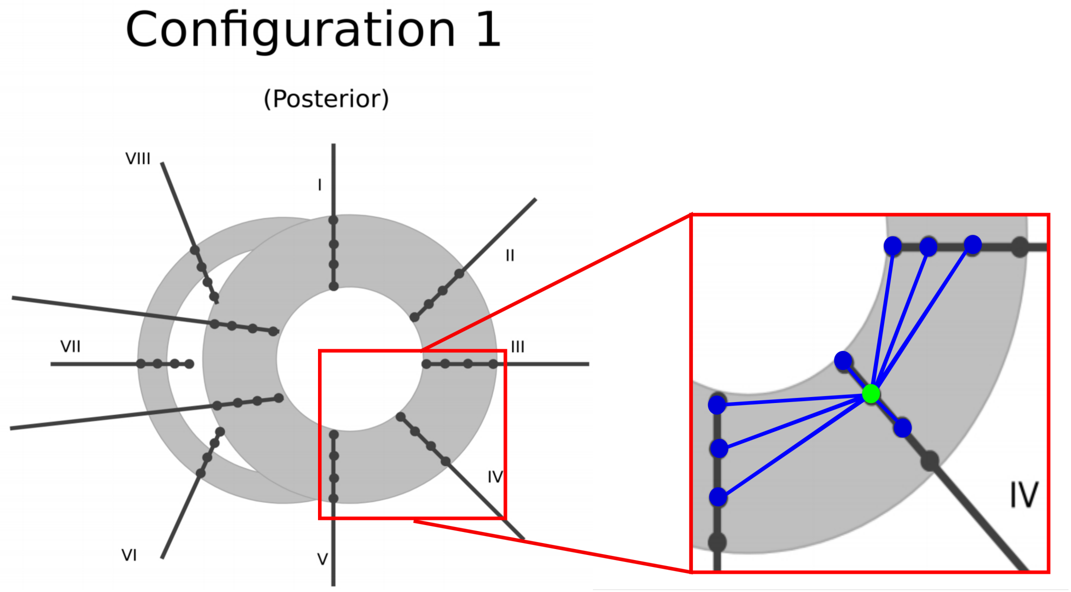

Figure S1. Example of the neighbours (blue) of a typical electrode (green) inside one layer of the ventricles. All electrodes that are positioned 1 layer above and 1 layer under the green and blue electrodes are also neighbours of the green electrode.

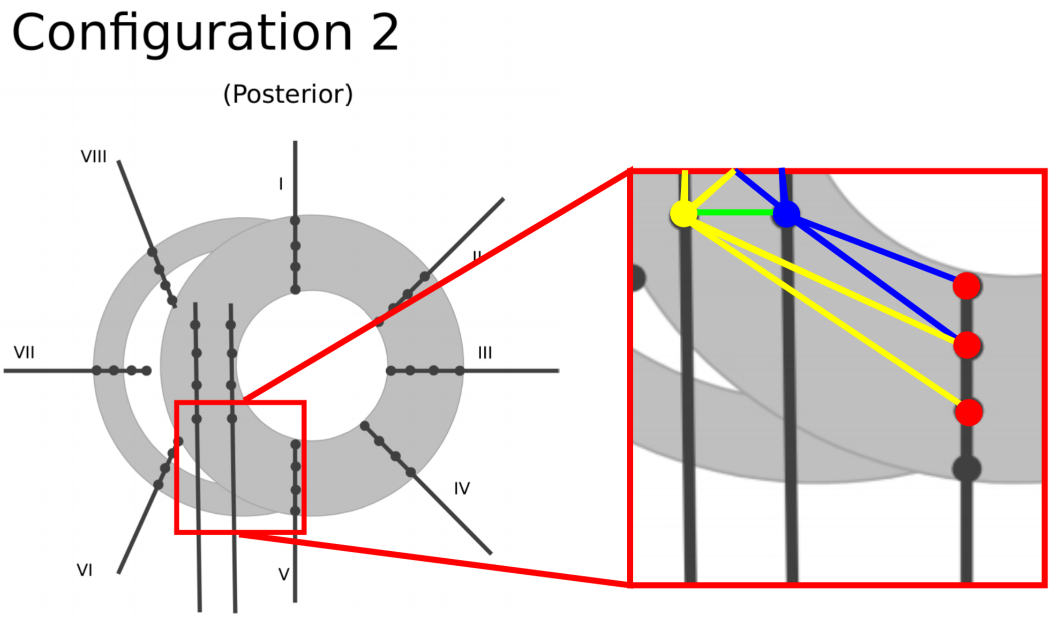

Figure S2. Example of the neighbouring algorithm in configuration 2 between the septum and right ventricle.

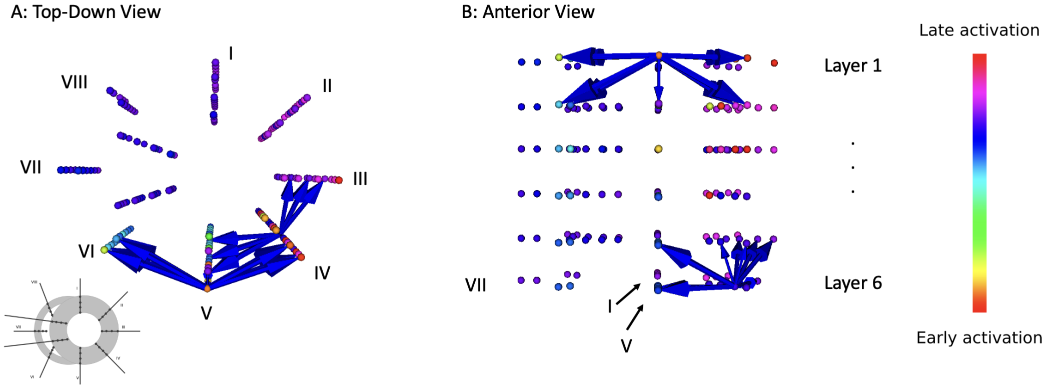

Figure S3. Example of two detected focal sources (origins of blue arrows). Activation time colour scale spans 200 ms.

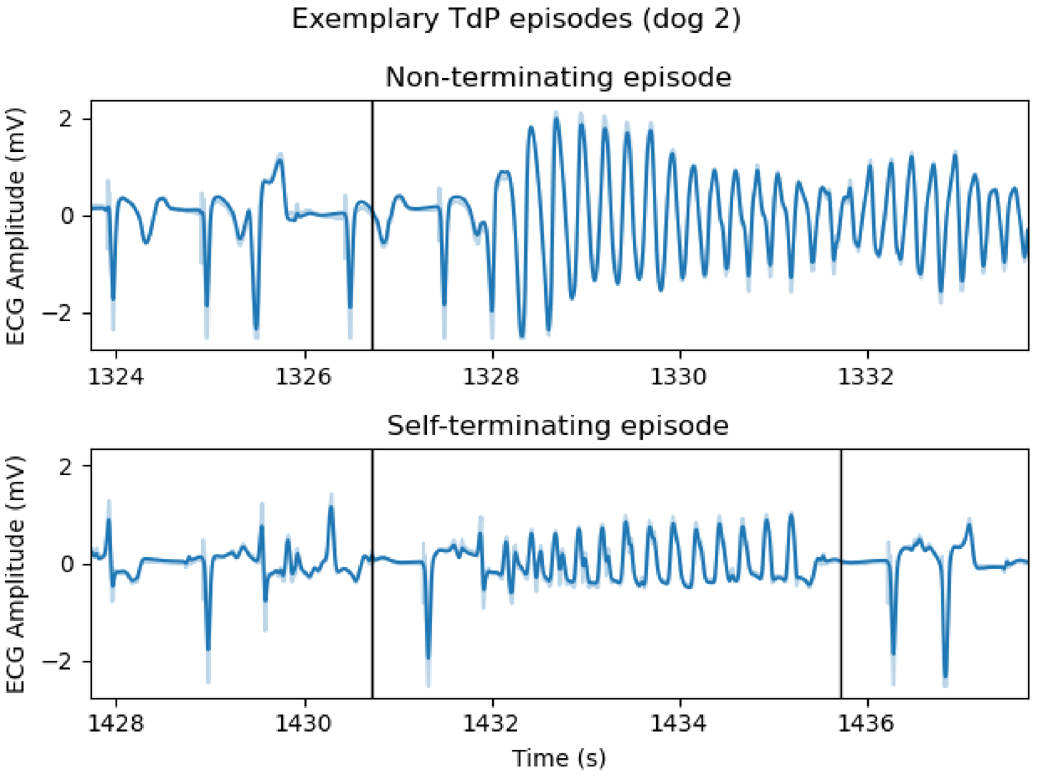

Figure S4. Exemplary ECG recordings from one dog. Unfiltered (light-blue) and filtered (blue) signals shown. Top shows the beginning of a typical non-terminating (NT) episode, bottom a typical self-terminating (ST) episode. Both signals exhibit the change in QRS morphology and amplitude typical for TdP.

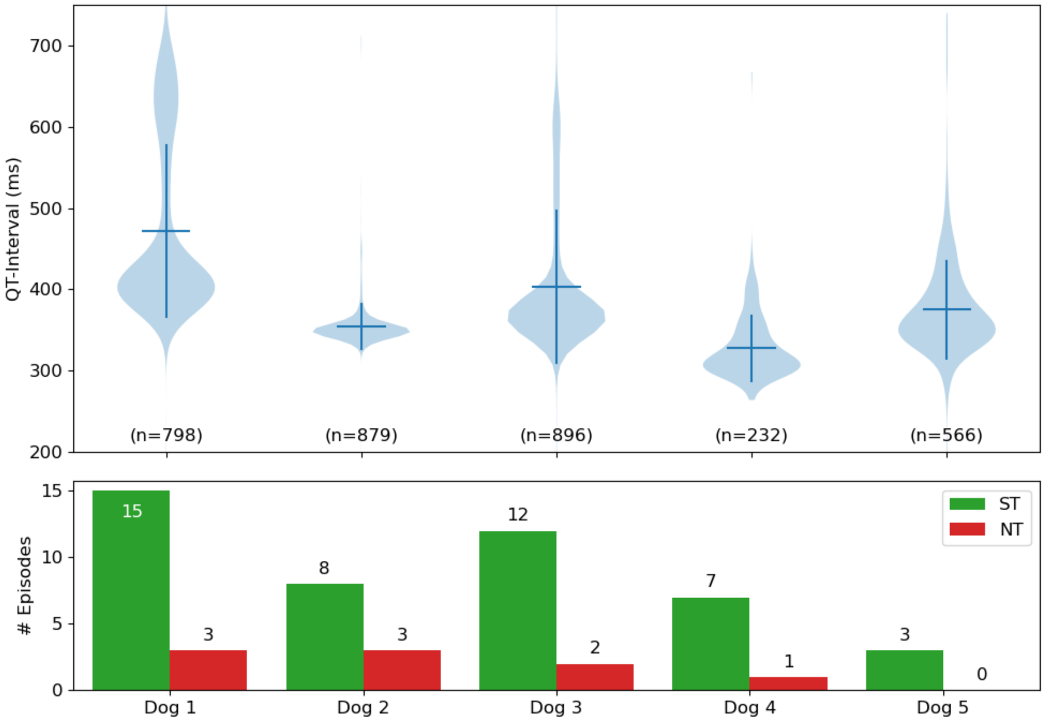

Figure S5. Distributions of QT intervals before the first TdP episodes (top; n is the number of beats measured) and number of self-terminating (ST) and non-terminating (NT) episodes recorded in individual animals. Correlation was tested between median QT interval and QT interval variance vs. total number of episodes and number of NT episodes in each dog. None of the four tests indicated correlation with the p-values ranging from 0.24 to 0.72 and the 95% confidence interval for Pearson's r covering more than 3/4 of the interval $[-1, 1]$ in all cases.

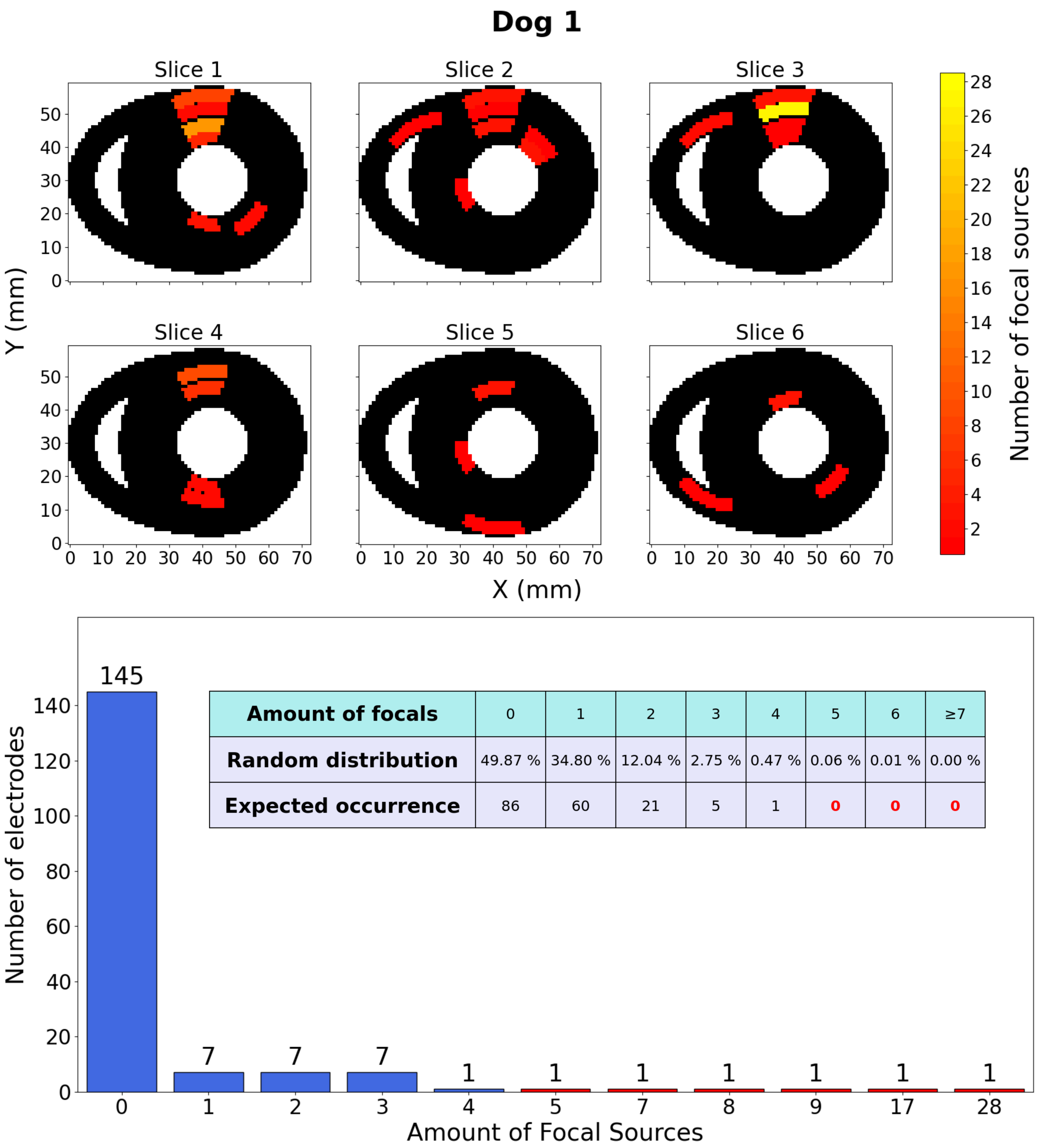

Amount of focals	0	1	2	3	4	5	6	≥7
Random distribution	49.87 %	34.80 %	12.04 %	2.75 %	0.47 %	0.06 %	0.01 %	0.00 %
Expected occurrence	86	60	21	5	1	0	0	0

Figure S6.

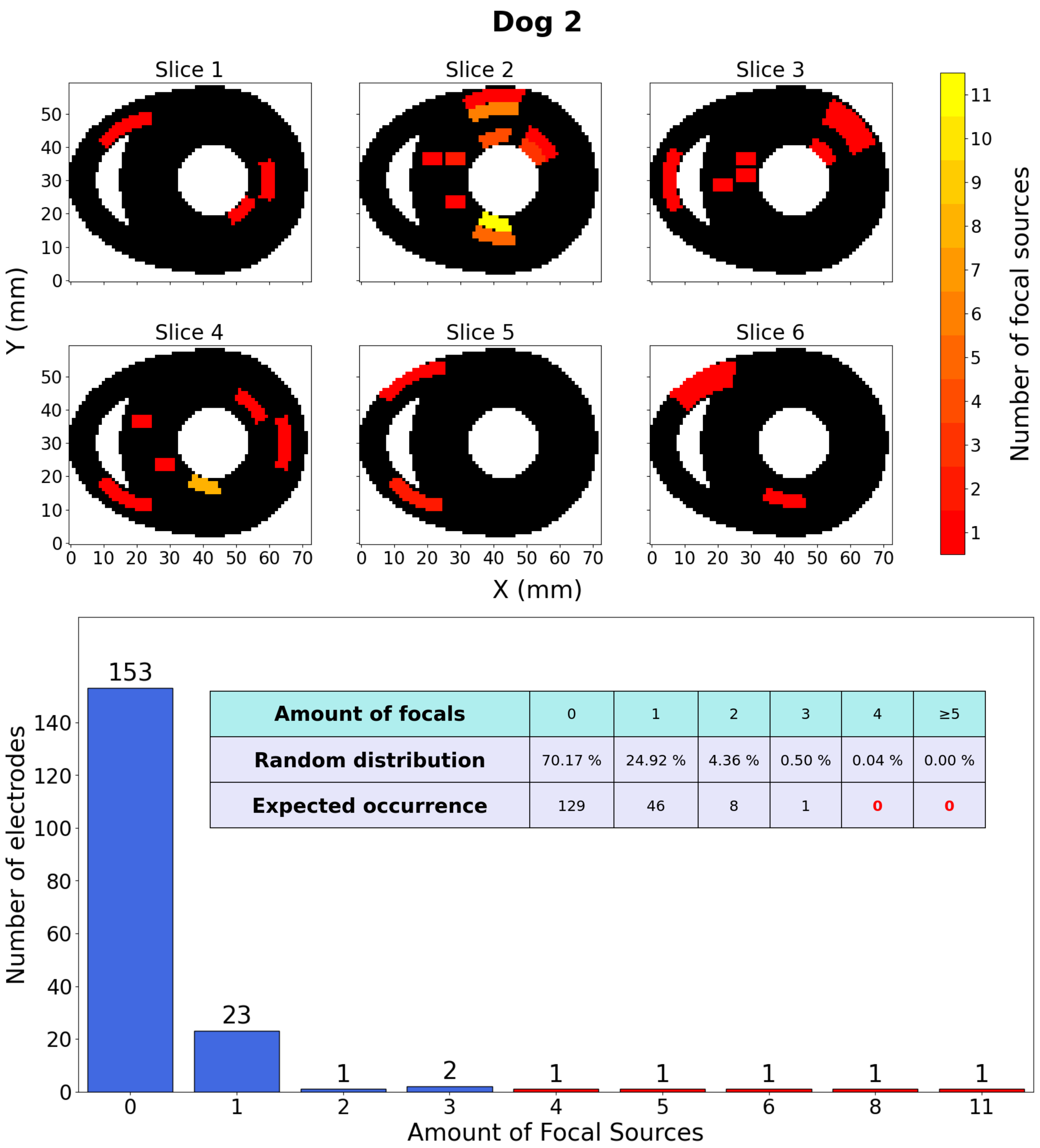

Amount of focals	0	1	2	3	4	≥5
Random distribution	70.17 %	24.92 %	4.36 %	0.50 %	0.04 %	0.00 %
Expected occurrence	129	46	8	1	0	0

Figure S7.

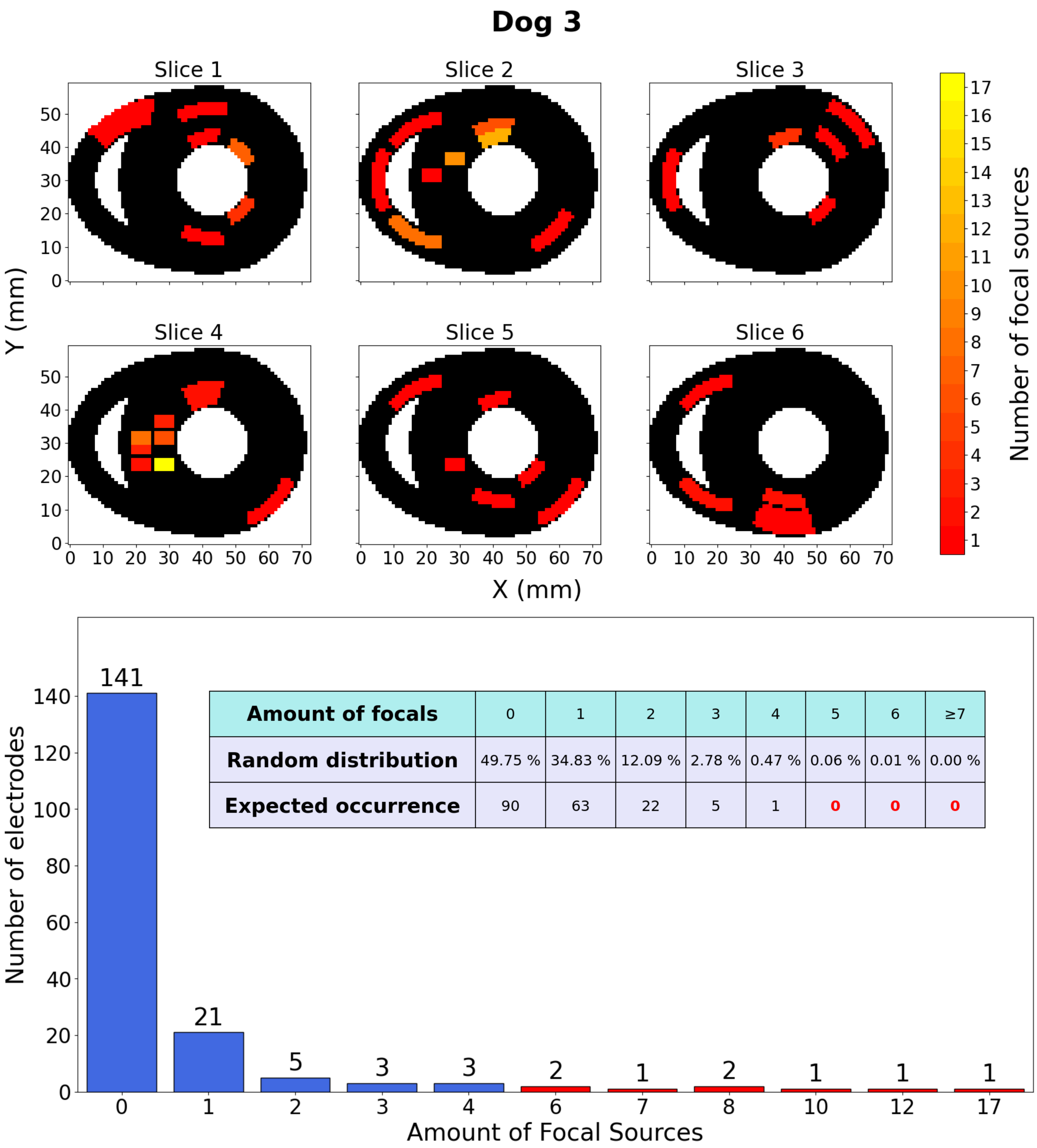

Amount of focals	0	1	2	3	4	5	6	≥7
Random distribution	49.75 %	34.83 %	12.09 %	2.78 %	0.47 %	0.06 %	0.01 %	0.00 %
Expected occurrence	90	63	22	5	1	0	0	0

Figure S8.

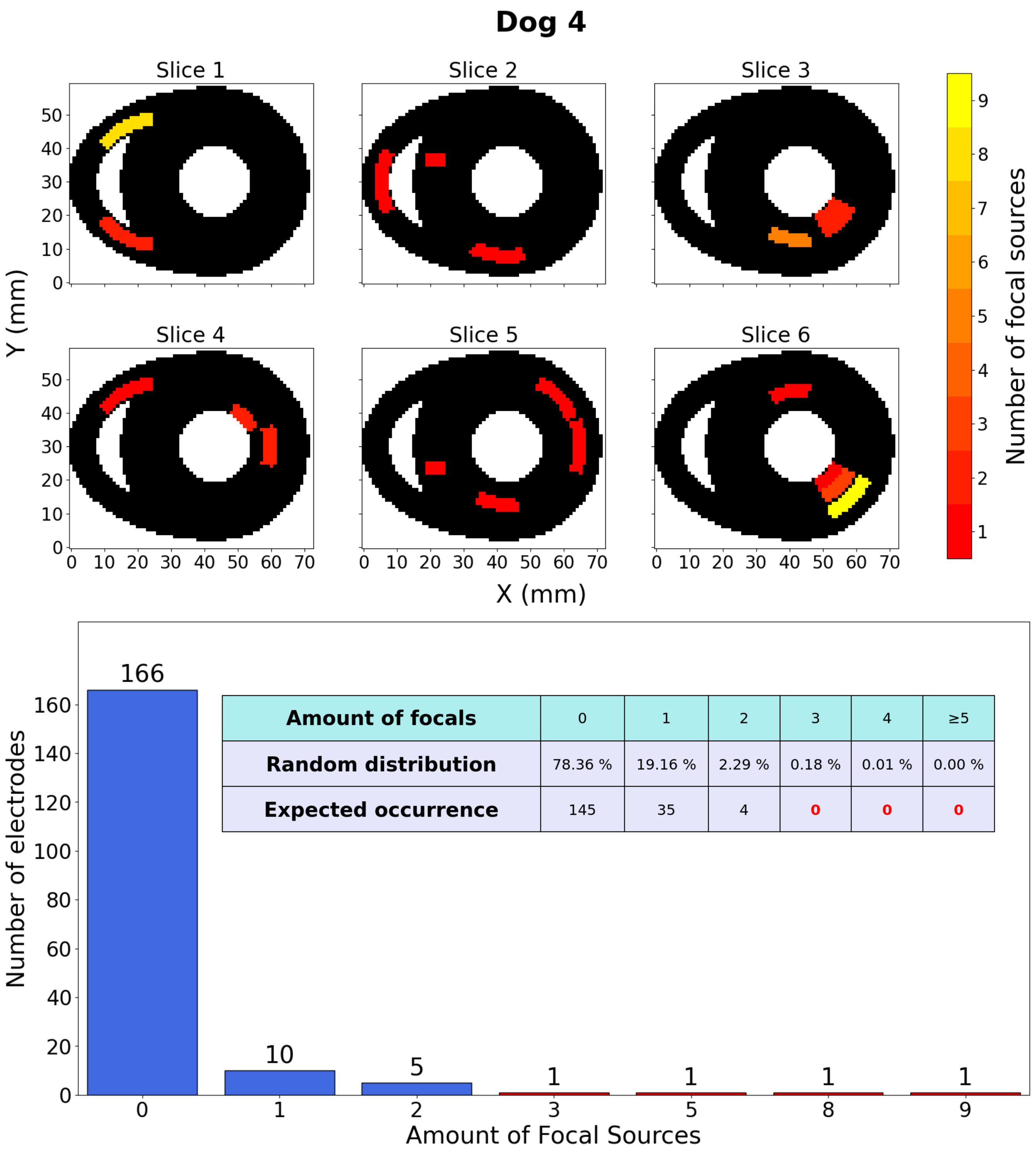

Amount of focals	0	1	2	3	4	≥5
Random distribution	78.36 %	19.16 %	2.29 %	0.18 %	0.01 %	0.00 %
Expected occurrence	145	35	4	0	0	0

Figure S9.

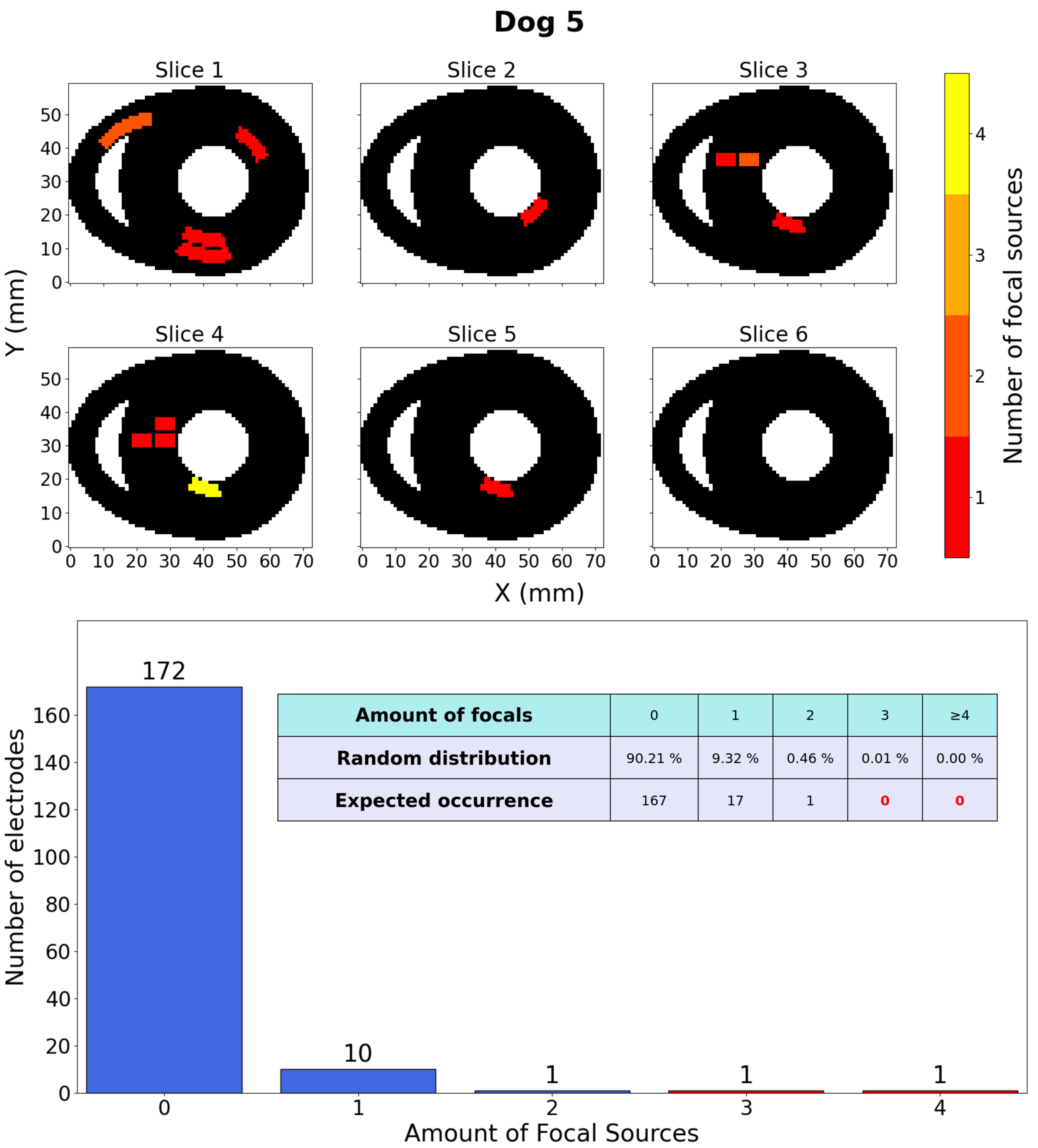
Dog 5
Slice 1
Slice 2
Slice 3
Slice 4
Slice 5
Slice 6
Y (mm)
X (mm)
Number of focal sources
Amount of focals | 0 | 1 | 2 | 3 | ≥4
Random distribution | 90.21 % | 9.32 % | 0.46 % | 0.01 % | 0.00 %
Expected occurrence | 167 | 17 | 1 | 0 | 0
172
10
1
1
1
Number of electrodes
Amount of Focal Sources

Figure S10.

REFERENCES

Van Nieuwenhuyse, E., Strisciuglio, T., Lorenzo, G., El Haddad, M., Goedgebeur, J., Van Cleemput, N., et al. (2021). Evaluation of directed graph-mapping in complex atrial tachycardias. *JACC: Clinical Electrophysiology* doi:https://doi.org/10.1016/j.jacep.2020.12.013
